# Effect of acetazolamide on visuomotor performance at high altitude in healthy people 40 years of age or older—RCT

**DOI:** 10.1371/journal.pone.0280585

**Published:** 2023-01-20

**Authors:** Aurelia E. Reiser, Michael Furian, Mona Lichtblau, Aline Buergin, Simon R. Schneider, Paula Appenzeller, Laura Mayer, Lara Muralt, Maamed Mademilov, Ainura Abdyraeva, Shoira Aidaralieva, Aibermet Muratbekova, Azamat Akylbekov, Ulan Sheraliev, Saltanat Shabykeeva, Talant M. Sooronbaev, Silvia Ulrich, Konrad E. Bloch

**Affiliations:** 1 Department of Respiratory Medicine, University Hospital Zurich, Zurich, Switzerland; 2 Swiss-Kyrgyz High Altitude Medicine and Research Initiative, Zurich, Switzerland/ Bishkek, Kyrgyz Republic; 3 Department of Respiratory Medicine, National Center of Cardiology and Internal Medicine, Bishkek, Kyrgyz Republic; Clinica Luganese Moncucco, SWITZERLAND

## Abstract

**Objective:**

Altitude travel is increasingly popular also for middle-aged and older tourists and professionals. Due to the sensitivity of the central nervous system to hypoxia, altitude exposure may impair visuomotor performance although this has not been extensively studied. Therefore, we investigated whether a sojourn at moderately high altitude is associated with visuomotor performance impairments in healthy adults, 40y of age or older, and whether this adverse altitude-effect can be prevented by acetazolamide, a drug used to prevent acute mountain sickness.

**Methods:**

In this randomized placebo-controlled parallel-design trial, 59 healthy lowlanders, aged 40-75y, were assigned to acetazolamide (375 mg/day, n = 34) or placebo (n = 25), administered one day before ascent and while staying at high altitude (3100m). Visuomotor performance was assessed at 760m and 3100m after arrival and in the next morning (post-sleep) by a computer-assisted test (Motor-Task-Manager). It quantified deviation of a participant-controlled cursor affected by rotation during target tracking. Primary outcome was the directional error during post-sleep recall of adaptation to rotation estimated by multilevel linear regression modeling. Additionally, adaptation, immediate recall, and correct test execution were evaluated.

**Results:**

Compared to 760m, assessments at 3100m with placebo revealed a mean (95%CI) increase in directional error during adaptation and immediate recall by 1.9° (0.2 to 3.5, p = 0.024) and 1.1° (0.4 to 1.8, p = 0.002), respectively. Post-sleep recall remained unchanged (p = NS), however post-sleep correct test execution was 14% less likely (9 to 19, p<0.001). Acetazolamide improved directional error during post-sleep recall by 5.6° (2.6 to 8.6, p<0.001) and post-sleep probability of correct test execution by 36% (30 to 42, p<0.001) compared to placebo.

**Conclusion:**

In healthy individuals, 40y of age or older, altitude exposure impaired adaptation to and immediate recall and correct execution of a visuomotor task. Preventive acetazolamide treatment improved visuomotor performance after one night at altitude and increased the probability of correct test execution compared to placebo.

**ClinicalTrials.gov identifier:**

ClinicalTrials.gov NCT03536520.

## Introduction

Mountain areas are the second most popular tourist destination worldwide [1]. Due to the sensitivity of the central nervous system to hypoxia, high altitude exposure can lead to cognitive impairment [2–4], endangering people working or performing leisure activities. Increases in reaction time and declines in attention, language, memory, and executive functions have been noticed mostly at altitudes over 3,500 m, within hours to days of exposure [5–10]. Yet, deterioration in visuomotor adaptation and learning have been reported at a mere altitude of 2,590 m [11].

Studies on effective medication against cognitive impairment at altitude are scant. Acetazolamide, a drug used in the prevention and treatment of acute mountain sickness (AMS), stimulates ventilation by inducing a metabolic acidosis via renal bicarbonate excretion and by tissue acidosis influencing central and peripheral chemoreceptors [12, 13]. The rise in ventilation induced by acetazolamide improves systemic and cerebral oxygenation [14, 15] and may therefore partially reverse the altitude-induced hypobaric hypoxia of the neuromuscular system and its clinical manifestations. Consistently, in patients with chronic obstructive pulmonary disease (COPD) travelling to 3,100 m, we found that preventive treatment with acetazolamide improved oxygenation, visuomotor performance, learning and postural control compared to placebo [16, 17]. In healthy individuals, the few studies on effects of acetazolamide on cognition at high altitude are inconclusive because of the small number of participants and methodological limitations [18, 19]. Animal experiments have suggested a potential role of different carbonic anhydrase modulators in memory processing [20–22].

Therefore, the current study aims to further investigate the effect of altitude and preventive acetazolamide treatment on cognitive performance in healthy individuals. We focused on visuomotor performance as this component of neurocognition is known to deteriorate even at moderately high altitudes, therefore being relevant for professionals and tourists alike. Furthermore, we studied people 40 years of age or older since more than half of all tourists belong to this age group [23]. We hypothesized that acetazolamide alleviates altitude-induced impairment of visuomotor performance compared to placebo in healthy individuals 40 years of age or older.

## Methods

### Study design

This randomized, double-blind, placebo-controlled, parallel trial evaluating the efficacy of preventive acetazolamide treatment on visuomotor performance in healthy middle-aged lowlanders during a stay at high altitude. Data were collected from June to July 2018, with recruitment and baseline data collection being carried out at 760 m in a university clinic (National Center of Cardiology and Internal Medicine, Bishkek, Kyrgyzstan) and subsequent measurements at high altitude being held at a high altitude clinic (Too Ashu, Kyrgyzstan) at 3,100 m.

This study was part of a larger trial investigating the effect of acetazolamide on the prevention of AMS in healthy individuals older than 40 years. Baseline characteristics as well as data on clinical outcomes and AMS have been reported previously [24], however data on visuomotor performance, the focus of the current study, have not been published before.

### Standard protocol approvals, registrations, and patient consents

Participants provided written informed consent and the study was approved by the ethics committee of the National Center of Cardiology and Internal Medicine in Bishkek, Kyrgyzstan (2018–10). The study is registered on ClinicalTrials.gov (Identifier: NCT03536520).

### Participants

Healthy men and women aged 40 to 75 years who responded to word-of-mouth propaganda or were known to the study staff were invited to participate in this study. Participants had to live below 800 m and be free of any disease or need of medication. Subjects reporting either heavy smoking (>20 cigarettes/day), a history of smoking >20 pack-years, cessation of smoking less than 10 years ago, regular use of alcohol or a known allergy to acetazolamide or other sulfonamides were excluded.

### Interventions

Participants were randomized to treatment with either acetazolamide capsules (125 mg) or identically looking placebo capsules, one in the morning and two in the evening with a total daily acetazolamide dose of 375 mg. Baseline measurements including assessment of visuomotor performance were performed at 760 m before starting the treatment. A few weeks after initial testing, participants were invited back to start treatment one day before ascent. They then travelled 3–5 hours by minibus to the high altitude clinic at 3,100 m, where they stayed for 48 hours.

### Assessments

**Fig 1:**
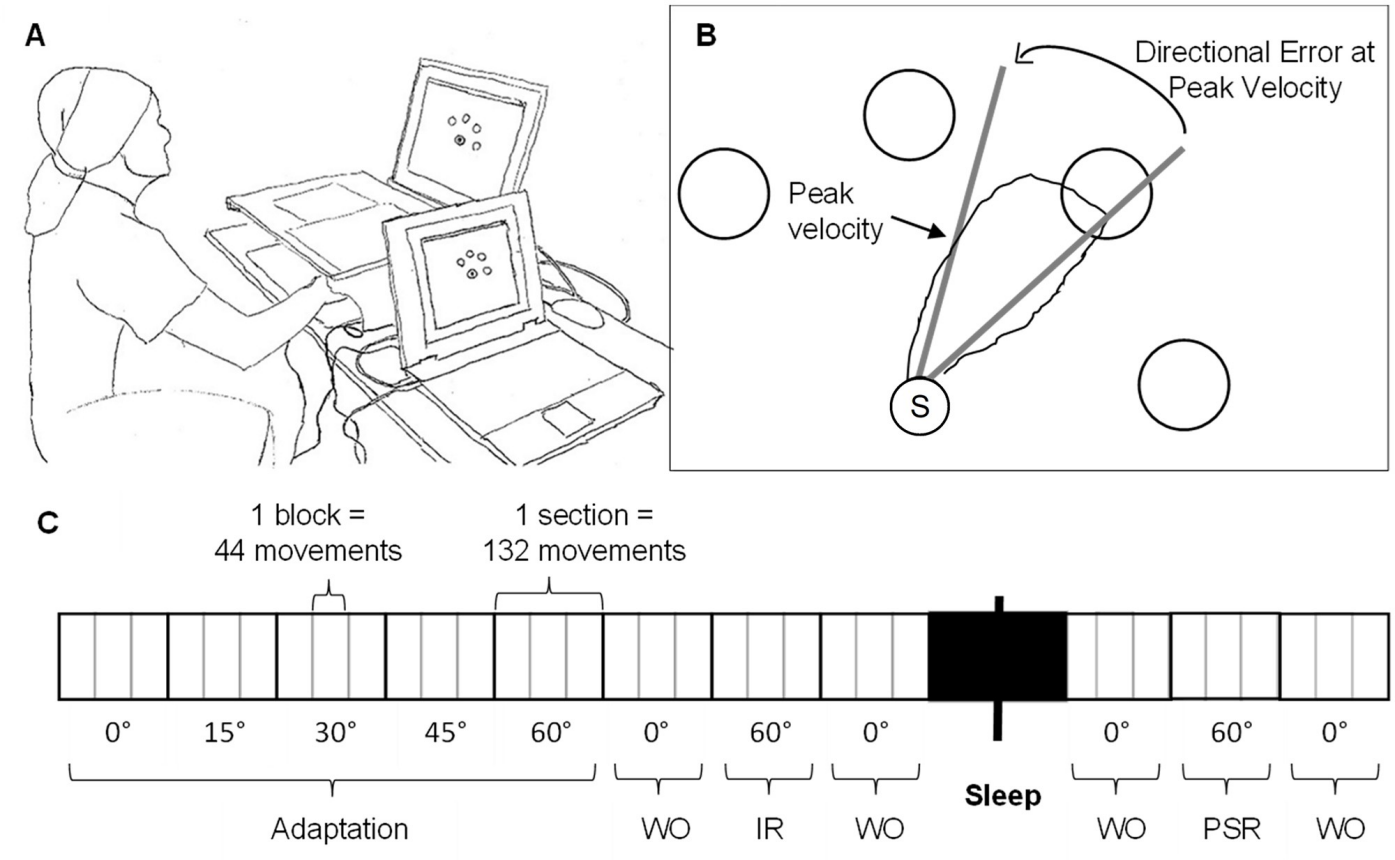
Motor task manager protocol. (A) Experimental setup of the motor task manager. Participants were seated in a quiet room in front of a computer screen displaying a central starting point and 4 peripheral targets, with the investigator observing the test on a separate screen positioned at a right angle to the participant. Hand and arm were concealed of the participant’s view, allowing no visual input regarding position and movement. (B) The directional error at peak velocity is the angle between the vector from the central starting point to the peripheral target and the vector from starting point (S) to the point of peak velocity during movement. (C) Protocol to test visuomotor adaptation and learning. Adaptation (A) and immediate recall (IR) were tested before sleep, post-sleep recall (PSR) the next morning after sleep. WO = washout.

Visuomotor performance was tested with the Motor Task Manager (MTM) test (ETT, Genova, Italy), a validated test shown to capture the effects of hypoxia on visuomotor learning and performance at altitude (see also Supporting information) [11]. Assessments were conducted within 4 hours after arrival and in the subsequent morning at baseline and at altitude, respectively. Intake of substances potentially influencing MTM performance such as alcohol or coffee were not available during the study, smoking was not permitted within 2 h before testing. The protocol was similar to that used in previous studies [11, 25] and is illustrated in Fig 1. Participants had to move a handheld cursor shielded from their view from a central starting point to one of four possible targets appearing pseudorandomized in the periphery of a computer screen. Without the participants being aware of it, the cursor position was rotated by the software in certain angles according to a predefined protocol. The protocol was divided into 11 test sections, with every section consisting of 3 blocks of tests with 44 movements each (132 movements per section). A short break was interposed between successive blocks. After a baseline section with 0° rotation, rotation was increased stepwise by 15° every section until reaching 60°. These first 5 sections were defined as the “adaptation to rotation” phase. Subsequently a washout block with 0° rotation was performed with the intention of deleting after-effects from short-term motor memory. In the subsequent section, termed “immediate recall”, participants were directly exposed to 60° rotation without prior adaption to lower rotation angles to test visuomotor learning. After another washout section, participants went to sleep. The next morning, subjects were re-exposed to 60° rotation in the “post-sleep recall” section to test consolidation of the previously learnt. As with immediate recall, this section was preceded and succeeded by a washout section.

Movements were evaluated according to predefined criteria and excluded if necessary (see Supporting information).

The direction of rotation was randomly assigned to the participant at 760 m and was subsequently inverted at 3,100 m to minimize the learning effect between first and second testing.

The main parameter of interest was the directional error (DE) at peak velocity, describing the angle between the vector from start to target and the vector from start to the point of maximal velocity of the outbound movement (See Fig 1B).

Additionally, clinical examinations, arterial blood gas analysis, spirometry, respiratory sleep studies and questionnaires assessing AMS (Lake Louise Score [26] and the Environmental Symptoms Questionnaire cerebral score [27]), sleep quality by a visual analog scale ranging from 0 (worst) to 100 (best) and daytime sleepiness (Karolinska sleepiness scale) were performed.

### Outcomes

The primary outcome was the DE at post-sleep testing. As this study is a nested trial, sample size calculation was performed for the primary outcome of the main trial, the incidence of AMS [24]. As there was no robust basis to perform a specific sample size estimation for visuomotor performance, as many participants of the main trial as logistically feasible were invited to undergo MTM tests.

Secondary outcomes included the analysis of the DE during immediate recall and 60° adaptation. To assess overnight learning, the differences in DE between post-sleep recall and adaptation respectively immediate recall were compared. Analogously, immediate learning was evaluated by the difference between adaptation and immediate recall. Furthermore, as a measure of correct test execution, the probability of performing valid movements and reaction time were analyzed.

### Randomization and blinding

Randomization was undertaken by a computer algorithm in a 1:1 allocation ratio to either acetazolamide or placebo, with minimization for age and sex. The participants undertaking MTM testing were randomly selected from the total study population participating the main trial. Participants and study investigators were blinded to drug allocation, and data analysists were blinded to drug and altitude intervention until data analysis was completed.

### Statistical analysis

Analysis was conducted in the per-protocol population due to the physiological nature of this study and for reasons of feasibility.

Mixed model linear regression with all available data was performed to analyze the effect of altitude and drug on DE and secondary outcomes such as laboratory values, vital parameters, or reaction time. If necessary, variable transformation was implemented to ensure normal distribution of residuals.

Besides including location and drug as independent variables, the model for DE also controlled for age, sex, target direction, number of valid movements and consecutive movement*block interaction effects over a section. In a stepwise forward selection approach oxygen saturation and mean arterial pressure were added as further factors.

To assess the overnight learning effect, a more complex model was developed including the 60° adaptation, immediate and post-sleep recall sections as independent variables.

The effects of altitude and medication on the probability of performing a valid movement were studied with a mixed logistic regression model developed using a stepwise forward selection approach. As this model showed a significant influence of drug and altitude on movement validity, movements excluded from DE analysis were not assumed to be missing at random. Hence, inverse probability weighting using the predicted result from the logistic regression was incorporated in the linear regression model for DE described above (See Supporting Information, S1–S3 Tables in S1 File). As parameter estimates from weighted and unweighted models were similar, unweighted models were used for further analysis as these estimates are more precise and standard errors are more reliable [28].

Data analysis was performed with Stata 14.2 (Stata Corp., College Station, Texas, USA). A p-value < 0.05 was considered as statistically significant.

## Results

**Fig 2:**
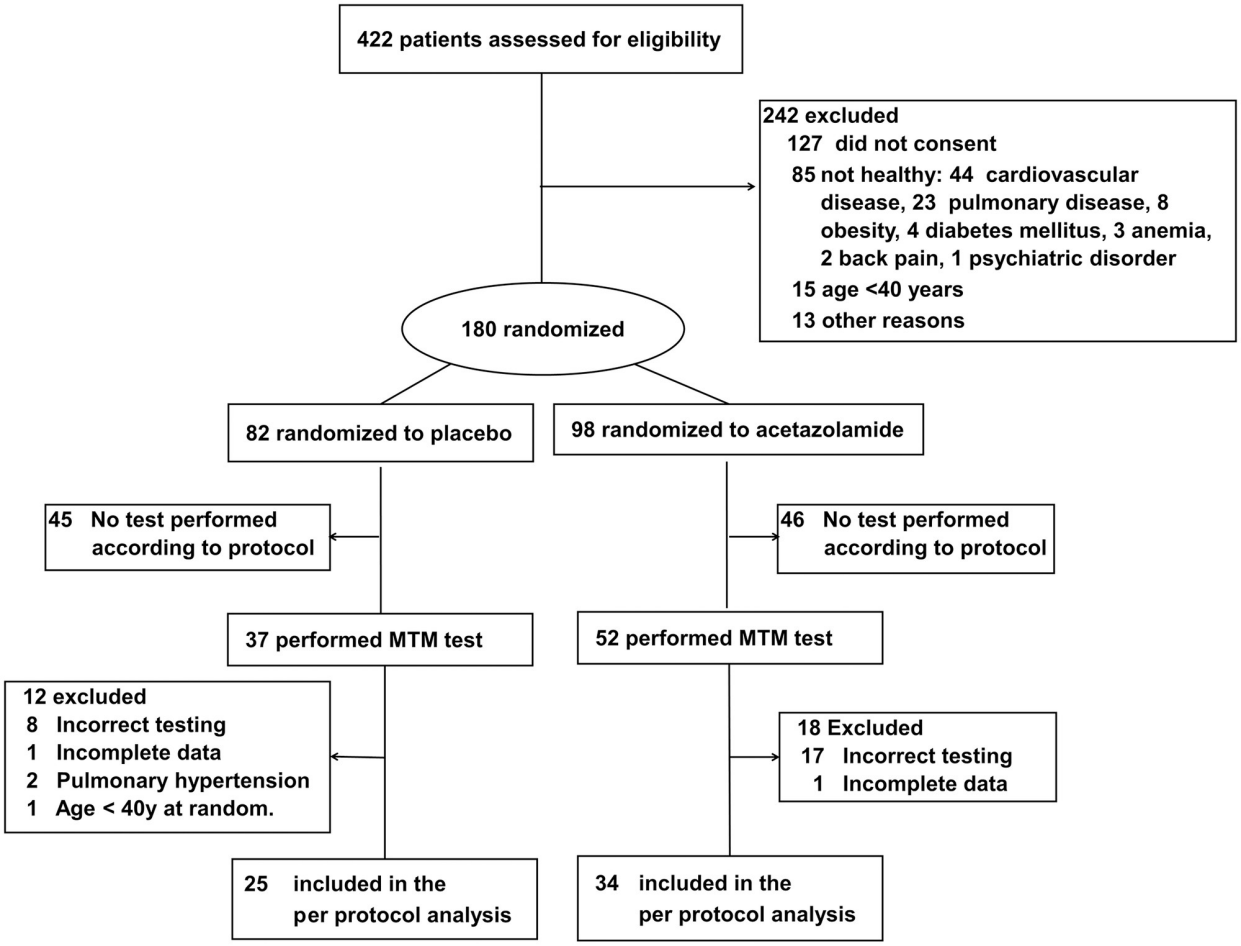
Participant flow chart. BMI = body mass index; COPD = chronic obstructive pulmonary disease; MTM = motor task manager; random. = randomization; y. = years.

The participant flow is shown in Fig 2. Of 422 people screened, 180 were randomized and participated in the main trial. Due to logistical reasons, a sample of 89 of the 180 participants underwent Motor Task Manager tests. Of these, 59 participants (25 assigned to placebo, 34 to acetazolamide) could be included in the per-protocol analysis.

No differences between participants assigned to placebo and acetazolamide were noticed at baseline (760m) (Table 1).

**Table 1:** Characteristics of participants at baseline (760m).

	Placebo	Acetazolamide	Total
**Number, n (%)**	25 (42.4%)	34 (57.6%)	59
**Female, n (%)**	13 (52.0%)	21 (61.8%)	34 (57.6%)
**Age, y**	52.9 ± 6.2	52.3 ± 7.0	52.6 ± 6.6
**Body Mass index, kg/m^2^**	27.3 ± 4.4	27.7 ± 3.8	27.5 ± 4.1
**Vital signs**			
**Mean arterial pressure, mmHg**	94.0 ± 8.7	92.3 ± 12.9	93.0 ± 11.2
**Heart rate, beats/min**	76 ± 11	81 ± 13	79 ± 13
**SpO_2,_ %**	96.0 ± 1.2	96.2 ± 1.4	96.1 ± 1.3
**Arterial blood analysis**			
**pH**	7.40 ± 0.02	7.40 ± 0.02	7.40 ± 0.02
**PaCO_2,_ kPa**	5.3 ± 0.4	5.2 ± 0.4	5.2 ± 0.4
**PaO_2_, kPa**	10.4 ± 0.9	10.1 ± 0.9	10.2 ± 0.9
**SaO_2_, %**	95.1 ± 1.3	94.7 ± 1.3	94.9 ± 1.3
**Bicarbonate concentration, mmol/l**	24.0 ± 1.7	23.9 ± 1.8	24.0 ± 1.8
**Lactate concentration, mmol/l**	1.1 ± 0.3	1.0 ± 0.3	1.1 ± 0.3
**Hemoglobin concentration, g/dl**	13.3 ± 1.5	13.9 ± 1.2	13.6 ± 1.3
**Hematocrit, %**	39.2 ± 4.4	40.8 ± 3.4	40.1 ± 3.9

Values are number (percent), mean ± SD. PaCO_2_ = arterial partial pressure of carbon dioxide; PaO_2_ = arterial partial pressure of oxygen; SaO2 = arterial oxygen saturation; SpO_2_ = pulse oximetry.

### Directional error at peak velocity

**Fig 3:**
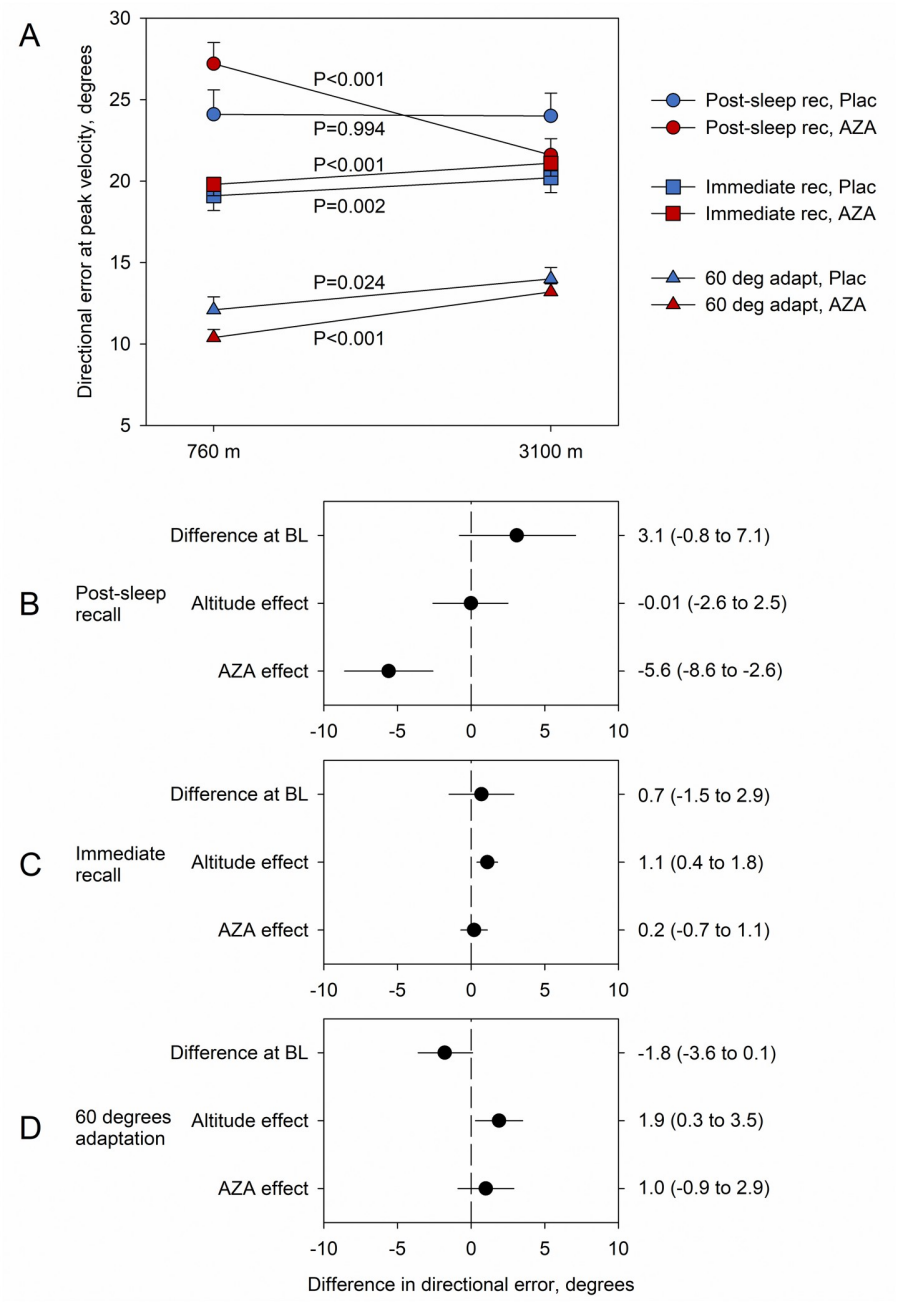
Effect of altitude and medication on directional error. (A) Average predicted margins of directional error at peak velocity during post-sleep recall (Post-sleep rec, circles), immediate recall (Immediate rec, squares) and 60° adaptation (60 deg adapt, triangles) in the groups using Placebo (Plac) and acetazolamide (AZA) at 760 m baseline and at 3100 m altitude. (B) Average marginal effects on directional error at peak velocity (with 95% confidence intervals) of groups at baseline (Difference at BL, without drug); (C) effects of altitude (Altitude effect); and (D) effects of acetazolamide at 3100 m (AZA effects).

As shown in Table 2 and Fig 3, participants taking placebo did not show a significant change of DE from 760 m to 3,100 m during post-sleep recall (mean difference -0.0°, 95%CI -2.6° to 2.5°, p = 0.994). However, in participants taking acetazolamide, the DE was significantly reduced by 5.6° (95%CI 4.1° to 7.1°, p < 0.001) at 3,100 m compared to 760 m resulting in a treatment effect of -5.6° (95%CI -8.6° to -2.6°, p < 0.001).

**Table 2:** Outcomes at low and high altitude (760 m and 3100 m).

	Placebo		Acetazolamide		Between-group difference	
					In altitude-induced change	
	760 m	3100 m	760 m	3100 m	Mean (95% CI)	p-Value
**Motor task manager outcomes**						
** **Directional error at peak velocity						
Post-sleep recall, °	24.1 ± 1.5	24.0 ± 1.4	27.2 ± 1.3	21.6 ± 1.0*	-5.6 (-8.6 to -2.6)	< 0.001
Immediate recall, °	19.1 ± 0.9	20.2 ± 0.9^†^	19.8 ± 0.7	21.1 ± 0.8*	0.2 (-0.7 to 1.2)	0.607
60° Adaptation, °	12.1 ± 0.8	14.0 ± 0.7^†^	10.4 ± 0.5	13.2 ± 0.5*	1.0 (-0.9 to 2.9)	0.317
** **Reaction time						
Post-sleep recall, ms	378 ± 19	374 ± 19	371 ± 16	334 ± 14*	-33 (-44 to -22)	< 0.001
Immediate recall, ms	375 ± 17	364 ± 17^†^	361 ± 14	345 ± 14*	-6 (-16 to 4)	0.278
60° Adaptation, ms	343 ± 18	344 ± 18	330 ± 15	317 ± 14*	-14 (-22 to -6)	0.001
** **Probability of performing a valid movement						
Post-sleep recall, %	63.1 ± 3.5	48.8 ± 4.0*	40.3 ± 2.7	62.0 ± 3.0*	36.0 (30.0 to 42.0)	< 0.001
Immediate recall, %	56.1 ± 4.0	51.7 ± 3.4	61.6 ± 2.9	57.8 ± 2.8	0.6 (-7.3 to 8.5)	0.887
60° Adaptation, %	67.8 ± 3.0	65.5 ± 3.1*	66.0 ± 2.7	67.9 ± 2.6*	4.2 (1.4 to 7.1)	0.003
**Vital parameters and arterial blood analyses**						
Mean arterial pressure, mmHg	94.6 ± 2.1	101.1 ± 2.1*	88.5 ± 1.8	90.9 ± 1.8	-4.2 (-8.7 to 0.2)	0.062
Heart rate, beats/min	67 ± 2	72 ± 2^†^	67 ± 2	69 ± 2	-2 (-6 to 2)	0.381
SpO_2_, %	96.4 ± 0.4	90.8 ± 0.4*	96.0 ± 0.3	92.8 ± 0.3*	2.4 (1.2 to 3.5)	< 0.001
pH	7.40 ± 0.004	7.43 ± 0.0*	7.40 ± 0.00	7.36 ± 0.00*	-0.07 (-0.08 to -0.05)	< 0.001
PaCO_2_, kPa	5.3 ± 0.1	4.7 ± 0.1*	5.2 ± 0.1	4.3 ± 0.1*	-0.4 (-0.6 to -0.3)	< 0.001
PaO_2_, kPa	10.4 ± 0.2	7.8 ± 0.2*	10.1 ± 0.1	8.6 ± 0.2*	1.2 (0.7 to 1.6)	< 0.001
SaO_2_, %	95.1 ± 0.4	87.6 ± 0.4*	94.7 ± 0.3	90.2 ± 0.4*	3.0 (1.8 to 4.1)	< 0.001
Bicarbonate, mmol/l	24.0 ± 0.3	22.8 ± 0.3*	23.9 ± 0.3	17.8 ± 0.3*	-4.9 (-5.7 to -4.1)	< 0.001
Hemoglobin concentration, g/dl	13.3 ± 0.3	13.7 ± 0.3^†^	13.8 ± 0.2	14.5 ± 0.2*	0.2 (-0.2 to 0.6)	0.303
Hematocrit, %	39.2 ± 0.8	40.4 ± 0.8^†^	40.8 ± 0.7	42.6 ± 0.7*	0.7 (-0.5 to 1.8)	0.256
**Questionnaire assessments**						
Lake Louise score	0.64 ± 0.27	0.44 ± 0.27	0.71 ± 0.23	0.53 ± 0.23	0.02 (-0.95 to 1.00)	0.962
AMSc score	0.09 ± 0.03	0.04 ± 0.03	0.05 ± 0.02	0.07 ± 0.02	0.07 (-0.02 to 0.16)	0.123
Karolinska sleepiness scale	3.2 ± 0.3	3.1 ± 0.3	3.7 ± 0.3	4.0 ± 0.3	0.4 (-0.8 to 1.6)	0.492
Subjective sleep quality, %VAS	65.5 ± 4.9	54.7 ± 4.9	63.2 ± 4.2	62.6 ± 4.2	10.1 (-5.4 to 25.7)	0.202

Mean ±standard error or mean difference (95% confidence interval) from mixed regression analysis. Motor task manager outcomes are shown before (adaption and immediate recall) and after sleep (post-sleep recall), other outcomes are presented only after sleep in the morning.

* p < 0.001;

^†^ p < 0.05.

AMSc = Acute Mountain Sickness–cerebral score [27]; PaCO_2_ = arterial partial pressure of carbon dioxide; PaO_2_ = arterial partial pressure of oxygen; Sa O_2_ = arterial oxygen saturation; SpO_2_ = pulse oximetry; VAS = visual analog scale ranging from 0 (worst) to 100 (best) sleep quality.

Contrary to post-sleep recall, exposure to altitude led to a significant increase in DE, both in immediate recall and 60° adaptation with a mean change of 1.1° (95%CI 0.4° to 1.8°, p = 0.002) and 1.9° (95%CI 0.3° to 3.5°, p = 0.024) in participants taking placebo. DE also increased in participants taking acetazolamide, with mean changes of 1.3° (95%CI 0.7° to 1.9°, p < 0.001) during immediate recall and 2.9° (95%CI 1.9° to 3.8°, p < 0.001) during 60° adaptation. The between-group differences in these altitude-induced changes in DE were not statistically significant (p > 0.05).

To examine the effect of altitude and acetazolamide on overnight learning, the differences of DE between post-sleep recall and 60° adaptation and immediate recall, respectively, were computed based on the models presented in S1-S3 Tables of S1 File. Correspondingly, the difference of DE between immediate recall and 60° adaptation was computed as a surrogate for immediate learning. Exposure to altitude led to an increase in all three differences in participants taking placebo (Difference immediate recall–adaptation 0.4°, 95% CI 0.2° to 0.5°, p < 0.001; post-sleep recall–adaptation 0.5°, 95% CI 0.3° to 0.7°, p < 0.001; post-sleep recall–immediate recall 0.1°, 95% CI 0.1° to 0.2°, p < 0.001). In contrast, participants treated with acetazolamide showed no significant differences in measures of overnight learning and immediate recall between 3,100 m and 760 m (post-sleep recall–adaptation 0.0°, 95% CI -0.2° to 0.1°, p = 0.654; post-sleep recall–immediate recall 0.0°, 95% CI -0.1° to 0.0°, p = 0.653; immediate recall–adaptation 0.0°, 95% CI -0.1° to 0.1°, p = 0.655). Hence, a significant treatment effect with acetazolamide could be shown for all three differences (immediate recall– 60° adaptation -0.4°, 95% CI -0.6° to -0.2°, p < 0.001; post-sleep recall– 60° adaptation -0.5°, 95% CI -0.8° to -0.3°, p < 0.001; post-sleep recall–immediate recall -0.2°, -0.2° to -0.1°, p < 0.001).

### Movement validity

**Fig 4:**
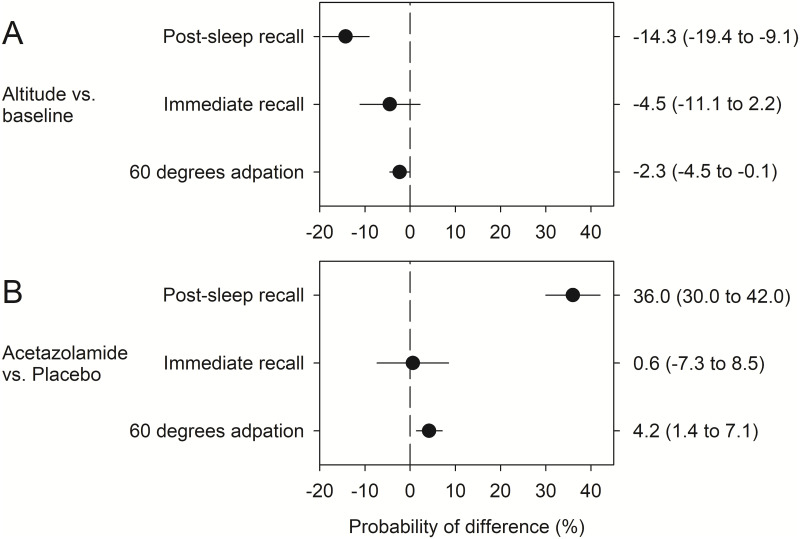
Effect of altitude and drug on probability of performing a valid movement. (A) Difference in the probability of performing a valid movement at altitude (3100 m) compared to baseline (760 m) during post-sleep recall, immediate recall and 60° adaptation under placebo. (B) Difference in altitude-induced change (3100 vs. 760 m) of the probability of performing a valid movement between acetazolamide and placebo. Results are displayed as average marginal effects with the corresponding 95% confidence intervals.

Altitude exposure deteriorated the capability of performing valid movements during post-sleep recall in participants taking placebo (reduction in chance of valid movement at 3,100 m by 14%, 95%CI 9% to 19%, p < 0.001) (Fig 4), while participants taking acetazolamide had a higher chance of a valid movement at 3,100 m (increase by 22%, 95%CI 19% to 25%, p < 0.001). A similar effect could be observed during 60° adaption with a reduction in chance of valid movement at 3,100 m with placebo by 2%, 95%CI 0% to 4%, p = 0.039; and with acetazolamide an increase in chance of valid movement at 3100 m by 2%, 95%CI 0% to 4%, p = 0.035. During immediate recall, no significant effect could be observed in either group. Therefore, the treatment effect with acetazolamide for performing a valid movement during post-sleep recall at 3,100 m vs. 760 m was 36% (95%CI 30% to 42%, p < 0.001) (Fig 4 and Supporting Information, S4 Table in S1 File). Effects were weaker for 60° adaption (4%, 95%CI 1% to 7%, p = 0.003) and nonsignificant for immediate recall (1%; 95%CI -7% to 8%, p = 0.887).

### Reaction time

No change of reaction time could be observed between 760 m and 3,100 m in participants taking placebo during post-sleep recall and 60° adaptation (Table 2). During immediate recall, reaction time decreased at altitude by 11 ms (95%CI 3 to 18, p = 0.007). In participants taking acetazolamide, reaction time decreased during all 3 measurements, with changes of -37 ms (95%CI -44 to -30, p < 0.001) for post-sleep recall, -16 ms (95%CI -23 to -10, p < 0.001) for immediate recall and -13 ms (95%CI -18 to -8, p < 0.001) for 60° adaptation. A significant treatment effect of acetazolamide could be observed during post-sleep recall and 60° adaptation with a between-group difference of altitude-induced change of -33 ms (95%CI -44 to -22, p < 0.001) and -14 ms (95%CI -22 to -6, p = 0.001), respectively. The difference was nonsignificant for immediate recall (-6 ms, 95%CI -16 to 4, p = 0.278).

### Vital signs, laboratory values and results of questionnaires

Arterial blood gas analysis showed normal results at 760 m. In participants taking placebo, an altitude-induced hypoxemia could be observed at 3,100 m. With acetazolamide, the altitude-induced hypoxemia was less pronounced. pH and PaCO_2_ were lower at 3,100 m compared to the placebo group indicating the expected development of metabolic acidosis under acetazolamide (Table 2).

A raise of heart rate and mean arterial pressure at 3,100 m could only be observed in the placebo group, the treatment effect was nonsignificant. No significant changes in scores assessing sleep quality and altitude related symptoms were noted.

### Influence of oxygen saturation, age, and sex on MTM performance

**Fig 5:**
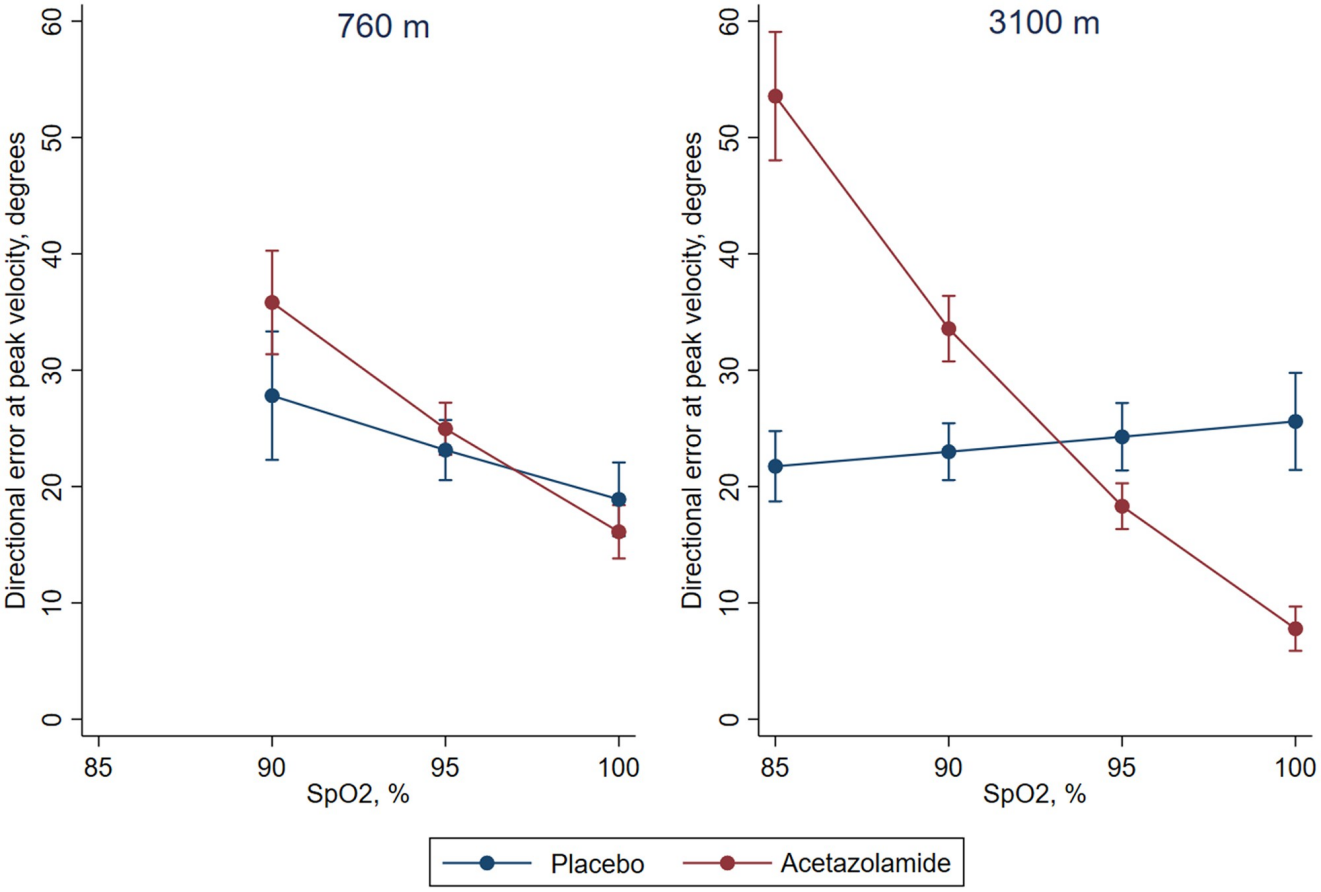
Effect of oxygen saturation on directional error. Average predicted margins of directional error at peak velocity as a function of pulse oximetry (SpO2) at 760 m baseline and at 3100 m altitude for patients taking placebo and acetazolamide, respectively.

Higher finger-tip oxygen saturation (SpO_2_) in the morning was associated with improved performance during post-sleep recall already at 760 m (DE -1.6°, 95%CI -2.0° to -1.1°, p < 0.001) and increased probability of a valid movement (2.1%, 95%CI 1.4% to 2.8%, p < 0.001). An exploratory analysis of post-sleep recall at 3,100 m in participants taking acetazolamide showed that whilst participants with a low oxygen saturation had a higher DE than comparable participants with placebo (SpO_2_ of 85%: 31.8°, 95% CI 25.6° to 38.1°, p < 0.001; SpO_2_ of 90%: 10.6°, 6.9° to 14.3°, p < 0.001), participants with a high oxygen saturation had a significantly lower DE (SpO_2_ of 95%: -6.0°, 95%CI -9.5° to -2.4°, p < 0.001; SpO_2_ of 100%: -17.8°, 95%CI -22.4° to -13.2°, p < 0.001) than corresponding participants with placebo (Fig 5).

Independent of block (post-sleep recall, immediate recall, 60° adaptation), for every increase in age by 1 year, the probability of a valid movement decreased by 1% (95%CI -2% to 0%, p = 0.003; 95%CI -2% to -1%, p < 0.001; 95%CI -2% to -1% p < 0.001 respectively). DE increased significantly with age (p = 0.005 for post-sleep recall, p = 0.008 for immediate recall, p = 0.001 for 60° adaptation).

Females had a lower chance of performing a valid movement compared to men, with values ranging from 18% in immediate recall (95%CI 25% to 10%, p < 0.001) to 10% in 60° adaptation (95%CI 18% to 3%, p = 0.009) and 9% in post-sleep recall (95%CI 19% to 0%, p = 0.048). Sex did not have a significant effect on DE with a difference in treatment effect between males and females of 0.0° (95%CI -0.4° to 0.5°, p = 0.857) in post-sleep recall, 0.0° (95%CI -0.0° to 0.0°, p = 0.6621) in immediate recall and 0.0° (95% CI -0.0° to 0.0°, p = 0.298) in 60° adaptation.

The study drugs were generally well tolerated and severe side effects did not occur. A detailed account on side effects in the main population of this trial has been published [24].

## Discussion

We performed a randomized, placebo-controlled, double-blind trial to investigate the effect of preventive treatment with acetazolamide on visuomotor performance in middle-aged individuals 40 years of age or older acutely exposed to high altitude. In participants taking placebo, performance during 60° adaptation and immediate recall was impaired at 3,100 m compared to 760 m, whilst post-sleep recall was not significantly affected. Treatment with acetazolamide improved performance during post-sleep recall, but not immediate recall and 60° adaptation. Additionally, worsening of correct test execution at altitude during adaptation and post-sleep recall was prevented by acetazolamide. These novel findings may have important implications for professionals and tourists travelling to high altitude.

### Effect of altitude on neurocognition

It has been previously shown in young, healthy individuals that altitude can lead to impairment of cognitive [2, 3, 8] and visuomotor performance. Our data corroborate and expand these findings by demonstrating in middle-aged people that DE during a motor task was increased on the day of arrival at 3,100 m vs. 760 m. When observing the development of the DE from adaptation to the immediate recall and post-sleep recall, participants maintained a higher DE at altitude than at baseline during the recall phases, suggesting that immediate and overnight learning were affected by altitude exposure. This is in concordance with the results of a randomized cross-over study by Tesler et al. [11]. However, the absolute DE after sleep at altitude was not significantly higher than that at baseline. We postulate that two counteracting effects of altitude and repetition influenced the change of performance in the MTM test from baseline to altitude, even though we tried to minimize this effect by inverting the direction of rotation at altitude [29]. As the effect of repetition on DE was predominantly evident during post-sleep recall, but not during immediate recall and 60° adaptation, we hypothesize that the effect of repetition was primarily based on improved overnight recall efficacy rather than on long-term memorized adaptation strategies.

### Effect of acetazolamide on neurocognition

In this study, it could be shown that acetazolamide leads to a significant improvement of DE at altitude compared to baseline during post-sleep recall, but not during immediate recall and 60° adaptation. As DE in participants taking acetazolamide remained stable between immediate and post-sleep recall, whilst the DE of participants taking placebo increased the findings are consistent with amelioration of memory consolidation at altitude by acetazolamide, especially during sleep.

Consistently, acetazolamide increased the chance of performing a valid movement at altitude compared to placebo, the effect being strongest for post-sleep recall and it reduced reaction time at altitude compared to placebo during post-sleep recall and 60° adaptation.

In patients with COPD participating in a similar study with the same test and same dosage of acetazolamide, we found not only a significant positive effect of acetazolamide on post-sleep but also on immediate recall, which was not the case in our data. Possibly, the effect of acetazolamide during immediate recall was more evident in patients with COPD than in healthy participants due to the more pronounced cerebral hypoxemia in the placebo group of COPD patients at altitude in combination with a reduced response of cerebral blood flow to hypoxemia [30] that was alleviated by acetazolamide [14, 15].

In studies evaluating performance in the paced auditorial serial addition test (PASAT) and auditorial memory testing, White et al. [18] reported improved performance with 500 mg of acetazolamide per day at 3,600 m. In contrast, Wang et al. [19] reported a worse performance in the PASAT, digit symbol substitution test and operation span task with 250 mg acetazolamide per day at 3651 m. Yet differences in study design and statistical analysis as well as lack of data describing adaptation to altitude such as oxygen saturation prevent direct comparisons to our data.

Tesler et al. [11] suggested that the observed increase of DE from immediate recall to post-sleep recall at altitude was due to the reduction in slow waves during sleep at altitude, impairing sleep related memory consolidation. They found no evidence that the altered breathing pattern at altitude accounted for the decline in cognitive performance. Therefore, a positive effect of acetazolamide on the amount of slow waves during sleep may explain in part for the effect of acetazolamide on DE during post-sleep recall. Additionally, the higher oxygen saturation with acetazolamide at altitude due to an increase of ventilation may have contributed to the positive effect on neurocognition. This hypothesis is supported by studies showing that acetazolamide can increase cerebral tissue oxygenation [14, 15] and improve sleep quality [15, 31] also at high altitude.

### The role of oxygen saturation on cognitive performance

Consistent with previous reports [31–33] we found that acetazolamide improved oxygen saturation at altitude compared to placebo (Table 2). Participants taking acetazolamide with a high oxygen saturation after sleep (≥ 93%) had a lower DE in post-sleep recall than participants with a comparable oxygen saturation taking placebo. However, participants taking acetazolamide with a low oxygen saturation (< 93%) performed worse than their matching counterparts taking placebo (Fig 5). The mechanisms underlying this intriguing observation remain elusive but may include differential effects of acetazolamide on cerebral blood flow and its autoregulation under the influence of varying degrees of hypoxemia and hypocapnia.

### Limitations of the study

This study focused on visuomotor performance and did not assess other neuropsychological functions such as language, short-time memory, and executive functions, since deteriorations of these functions have been seldomly detected at altitudes around 3,000 m. Worsening of visuomotor performance on the other hand have been observed below 3,000 m [11], and thus at altitudes often reached by tourists.

It is known that savings of adaptation to visuomotor rotation can be observed not only days, but also months after previous testing, with the DE decreasing with every repetition of the test [34]. Due to logistical reasons all participants had to be tested first at 760 m and then at 3,100 m. We tried to minimize the effect of test repetition by inverting the direction of rotation at altitude as it is known that learning of counterrotation can prevent a substantial decrease of DE in relearning [29]. Despite this effort, our results suggest that two counteracting effects of altitude and repetition influenced the change of performance in the MTM test from baseline to altitude. This design unfortunately did not allow to fully distinguish between the effect of altitude and test repetition. Nevertheless, the effect of acetazolamide could be evaluated, as all participants independent of drug allocation were exposed to the same influence of repetition and altitude.

AMS scores were low in both groups. Therefore, no inferences regarding the incidence of AMS, effect of acetazolamide and neurocognitive performance could be drawn. On the other hand, this study illustrates that neurocognitive deteriorations can be measured before symptomatic AMS can be detected.

The participants of the current study were of predominantly urban origin, slightly overweight, most likely reflecting their sedentary lifestyle, and familiar with the use of electronic devices. It remains uncertain whether people from another socio-economic background would perform differently in MTM tests. The lack of sleep EEG recordings prevented a further analysis of the effect of acetazolamide on sleep structure and its correlation with neurocognitive performance.

## Conclusion

The preventive treatment with 375 mg acetazolamide per day significantly improved post-sleep visuomotor performance impairment at altitude in healthy, middle-aged people compared to placebo but had no effect on performance during adaptation and immediate recall. Acetazolamide also improved visuomotor learning and prevented worsening of correct test execution at altitude during adaptation and post-sleep recall. Our findings are clinically important as they illustrate potentially serious risks for mountaineers, and people working at altitude that may prevail even in the absence of warning symptoms of acute mountain sickness, but which can be improved by preventive acetazolamide treatment.

## Supporting information

S1 File (PDF)

S1 Dataset (ZIP)
