# Supplementary material for: Effect of acetazolamide on visuomotor performance at high altitude in healthy people 40 years of age or older—RCT
Source: PLoS One. 2023 Jan 20;18(1):e0280585. doi: 10.1371/journal.pone.0280585 (PMC9858039; doi:10.1371/journal.pone.0280585)
Supplement: S1 File — (PDF) [file pone.0280585.s001.pdf]

Supplement to

Effect of Acetazolamide on Visuomotor Performance at High Altitude in Healthy People Older than 40 Years - RCT

Aurelia Reiser [1,3], MmS, Michael Furian [1,3], PhD, Mona Lichtblau[1,3], MD, Aline Buergin[1,3], MD, Simon R. Schneider[1,3], MS, Paula Appenzeller[1,3], BM, Laura Mayer[1,3], MD, Lara Muralt[1,3], MD, Maamed Mademilov [2,3] , MD, Ainura Abdyraeva[2,3], MD, Shoira Aidaralieva[2,3], MD, Aibermet Muratbekova[2,3], MD, Azamat Akylbekov[2,3], MD, Ulan Sheraliev[2,3], MD, Saltanat Shabykeeva[2,3], MD, Talant M. Sooronbaev[2,3] , MD, Silvia Ulrich[1,3], MD, Konrad E. Bloch[1,3], MD.

[1]Dept. of Respiratory Medicine, University Hospital Zurich, Switzerland

[2]Dept. of Respiratory Medicine, National Center of Cardiology and Internal Medicine, Bishkek, Kyrgyz Republic

[3]Swiss-Kyrgyz High Altitude Medicine and Research Initiative, Zurich, Switzerland, Bishkek, Kyrgyz Republic

Correspondence:

Konrad E. Bloch, MD

University Hospital Zurich, Dept. of Respiratory Medicine

Raemistrasse 100, CH-8091 Zurich, Switzerland

konrad.bloch@usz.ch

Supplement to Methods

The motor task manager test (MTM)

The MTM evaluates the complex interplay among the visual system, central nervous processing and motor execution. It has been widely used in neurophysiological research. The directional error (DE) selected as the primary outcome in the current study, is commonly applied to study the influence of rotation in visuomotor interaction [1-3]. It has been shown to decrease in a double exponential manner after first exposure to rotation in multiple studies [1-3]. We employed the protocol described by Huber et al. [4] has shown that the decrease in DE from first to last movement under influence of rotation in post-sleep recall is correlated with an increase in slow wave activity during sleep in Brodmann areas 7 and 40. PET-imaging studies have shown that the posterior parietal cortex (Brodmann area 7) is indeed involved in adaptation to rotation [2, 5]. Furthermore, we have previously shown that deterioration of adaptation to rotation in post-sleep recall at high vs. low altitude is correlated to slow wave activity in the EEG [6]. The principles of using counter-rotation and washouts between sections have been discussed previously [3] and have been applied in the current study as well. It is known that the directional error is influenced by target direction and the starting position of the hand [7]. Even though test protocols were the same for each participant and each target appeared equally during a section, we integrated this factor into our regression models to account for this effect (Supplement Tables 1-4, parameter Target Index).

1. Krakauer JW, Pine ZM, Ghilardi MF, Ghez C. Learning of visuomotor transformations for vectorial planning of reaching trajectories. J Neurosci. 2000;20(23):8916-24. Epub 2000/01/11. doi: 10.1523/jneurosci.20-23-08916.2000. PubMed PMID: 11102502; PubMed Central PMCID: PMCPMC6773094.
2. Krakauer JW, Ghilardi MF, Mentis M, Barnes A, Veytsman M, Eidelberg D, et al. Differential cortical and subcortical activations in learning rotations and gains for reaching: a PET study. J Neurophysiol. 2004;91(2):924-33. Epub 2003/10/03. doi: 10.1152/jn.00675.2003. PubMed PMID: 14523069.
3. Krakauer JW, Ghez C, Ghilardi MF. Adaptation to visuomotor transformations: consolidation, interference, and forgetting. J Neurosci. 2005;25(2):473-8. doi: 10.1523/JNEUROSCI.4218-04.2005. PubMed PMID: 15647491; PubMed Central PMCID: PMCPMC6725486.
4. Huber R, Felice Ghilardi M, Massimini M, Tononi G. Local sleep and learning. Nature. 2004;430(6995):78-81. doi: 10.1038/nature02663.
5. Ghilardi M, Ghez C, Dhawan V, Moeller J, Mentis M, Nakamura T, et al. Patterns of regional brain activation associated with different forms of motor learning. Brain Res. 2000;871(1):127-45. doi: 10.1016/s0006-8993(00)02365-9. PubMed PMID: 10882792.
6. Tesler N, Latshang TD, Lo Cascio CM, Stadelmann K, Stoewhas AC, Kohler M, et al. Ascent to moderate altitude impairs overnight memory improvements. Physiol Behav. 2015;139:121-6. doi: 10.1016/j.physbeh.2014.11.033. PubMed PMID: 25449393.
7. Ghilardi MF, Gordon J, Ghez C. Learning a visuomotor transformation in a local area of work space produces directional biases in other areas. Journal of Neurophysiology. 1995;73(6):2535-9. doi: 10.1152/jn.1995.73.6.2535. PubMed PMID: 7666158.

Exclusion criteria for movements executed in the motor task manager test

- Start of movement > 1 cm away from predefined starting point
- Movement heads to wrong peripheral target
- Movement does not return to starting point
- Reaction time < 100 ms
- Point of maximal velocity is reached >1 s after appearance of target
- Movement too short (half path length <3 cm)
- Segmented movements with multiple velocity maxima

Supplementary Table S1: Linear regression for post-sleep recall - comparison of inverse probability weighted model versus unweighted model

**Post-sleep recall (square root of DE)**	**Weighted model**		**Unweighted model**	
	**Coefficient (95%CI)**	**p**	**Coefficient (95% CI)**	**p**
Acetazolamide vs. placebo	10.59 (-9.10 to 30.27)	0.292	10.27 (1.52 to 19.01)	0.021
3100 m. vs. 760 m.	-11.58 (-24.87 to 1.74)	0.088	-11.37 (-18.02 to -4.73)	0.001
Treatment effect (interaction drug-altitude)	21.09 (2.88 to 39.31)	0.023	20.84 (12.19 to 29.49)	< 0.001
Target direction (right to left)				
Target 2 vs. Target 1	-0.22 (-0.34 to -0.09)	0.001	-0.22 (-0.29 to -0.14)	< 0.001
Target 3 vs. Target 1	-0.66 (-0.84 to -0.49)	< 0.001	-0.67 (-0.74 to -0.59)	< 0.001
Target 4 vs. Target 1	-0.31 (-0.49 to -0.14)	< 0.001	-0.31 (-0.39 to -0.24)	< 0.001
Movement number (per additional movement)	-0.06 (-0.06 to -0.05)	< 0.001	-0.06 (-0.06 to -0.05)	< 0.001
Block				
Block 2 vs. Block 1	-1.74 (-2.00 to -1.48)	< 0.001	-1.74 (-1.89 to -1.59)	< 0.001
Block 3 vs. Block 1	-2.4 (-2.63 to -2.09)	< 0.001	-2.35 (-2.5 to -2.2)	< 0.001
Movement number per block				
Block 2 vs. Block 1	0.03 (0.02 to 0.04)	< 0.001	0.03 (0.02 to 0.04)	< 0.001
Block 3 vs. Block 1	0.04 (0.03 to 0.05)	< 0.001	0.04 (0.03 to 0.04)	< 0.001
Number of valid movements per block (per valid movement)	-0.02 (-0.03 to -0.01)	< 0.001	- 0.02 (- 0.03 to -0.02)	< 0.001
Age (per year)	0.04 (0.02 to 0.06)	0.001	0.04 (0.01 to 0.06)	0.005
Sex(female vs. male)	-0.04 (-0.45 to 0.37)	0.851	-0.03 (-0.37 to 0.31)	0.857
Mean arterial pressure (per 1 mmHg increase)	0.01 (-0.00 to 0.03)	0.160	0.01 (0.01 to 0.02)	< 0.001
Oxygen saturation (per 1% increase)	-0.1 (-0.27 to 0.07)	0.264	-0.09 (-0.17 to -0.02)	0.012
Oxygen saturation, acetazolamide vs.placebo	-0.11 (-0.31 to 0.09)	0.293	-0.11 (-0.2 to -0.02)	0.022
Oxygen saturation, 3100 vs. 760	0.12 (0.31 to 0.09)	0.080	0.12 (0.05 to 0.19)	0.001
Oxygen saturation, effect on treatment effect	-0.23 (-0.42 to -0.04)	0.017	-0.23 (-0.32 to -0.14)	< 0.001
Constant	14.1 (-2.6 to 30.8)	0.1	13.8 (6.41 to 21.12)	< 0.001
Random effects				
ID	0.4 (0.26 to 0.62)		0.39 (0.27 to 0.57)	
Residual	1.49 (1.37 to 1.62)		1.49 (1.45 to 1.54)	

DE = directional error at peak velocity

Supplementary Table S2: Linear regression for immediate recall - comparison of inverse probability weighted model versus unweighted model

Immediate recall (Square root of DE)	Weighted model		Unweighted model	
	**Coefficient (95%CI)**	**p**	**Coefficient (95% CI)**	**p**
Acetazolamide vs. placebo	0.08 (-0.2 to 0.36)	0.563	0.08 (-0.17 to 0.34)	0.522
3100 m. vs. 760 m.	0.12 (-0.15 to 0.4)	0.378	0.12 (0.05 to 0.2)	0.002
Treatment effect (Interaction drug-altitude)	0.02 (-0.34 to 0.38)	0.897	0.02 (-0.08 to 0.13)	0.646
Target direction (right to left)				
Target 2 vs. Target 1	-0.16 (-0.29 to -0.04)	0.012	-0.16 (-0.23 to -0.09)	< 0.001
Target 3 vs. Target 1	-0.59 (-0.75 to -0.43)	< 0.001	-0.59 (-0.66 to -0.52)	< 0.001
Target 4 vs. Target 1	-0.32 (-0.47 to -0.18)	< 0.001	-0.32 (-0.4 to -0.25)	< 0.001
Movement number (per additional movement)	-0.05 (-0.06 to -0.05)	< 0.001	-0.05 (-0.06 to -0.05)	< 0.001
Block				
Block 2 vs. Block 1	-1.75 (-1.98 to -1.53)	< 0.001	-1.75 (-1.9 to -1.6)	< 0.001
Block 3 vs. Block 1	-2.4 (-2.69 to -2.12)	< 0.001	-2.4 (-2.55 to -2.25)	< 0.001
Movement number per block				
Block 2 vs. Block 1	0.03 (0.03 to 0.04)	< 0.001	0.03 (0.03 to 0.04)	< 0.001
Block 3 vs. Block 1	0.04 (0.04 to 0.05)	< 0.001	0.04 (0.04 to 0.05)	< 0.001
Number of valid movements per block (per valid movement)	-0.03 (-0.04 to -0.01)	< 0.001	-0.03 (-0.03 to -0.02)	< 0.001
Age (per year)	0.03 (0.01 to 0.04)	0.002	0.03 (0.01 to 0.05)	0.008
Sex (female vs. male)	-0.19 (-0.47 to 0.09)	0.189	-0.19 (-0.44 to 0.07)	0.146
Constant	6.25 (5.18 to 7.31)	< 0.001	6.25 (5.16 to 7.35)	< 0.001
Random effects				
ID	0.33 (0.14 to 0.34)		0.21 (0.15 to 0.32)	
Residual	1.42 (1.29 to 1.55)		1.42 (1.38 to 1.46)	

DE = directonal error at peak velocity

Supplementary Table 3: Linear regression for 60° adaptation - comparison of inverse probability weighted vs. unweighted model

60° Adaptation (Sqrt of Sqrt of DE)	Weighted model		Unweighted model	
	**Coefficient (95%CI)**	**p**	**Coefficient (95% CI**	**p**
Acetazolamide vs. placebo	-2.91 (-9.8 to 3.98)	0.407	-2.96 (-5.2 to -0.72)	0.01
3100 m. vs. 760 m.	-0.64 (-5.99 to 4.71)	0.816	-0.71 (-2.52 to 1.1)	0.441
Treatment effect (Interaction drug-altitude)	5.29 (-2.25 to 12.82)	0.169	5.4 (3.13 to 7.67)	< 0.001
Target direction (right to left)				
Target 2 vs. Target 1	0.00 (-0.02 to 0.03)	0.706	(-0.01 to 0.02)	0.613
Target 3 vs. Target 1	-0.09 (-0.13 to -0.06)	< 0.001	-0.09 (-0.11 to -0.08)	< 0.001
Target 4 vs. Target 1	-0.02 (-0.05 to 0.02)	0.297	-0.02 (-0.04 to -0.00)	0.049
Movement number (per additional movement)	-0.01 (-0.01 to -0.00)	< 0.001	-0.01 (-0.01 to -0.00)	< 0.001
Block				
Block 2 vs. Block 1	-0.14 (-0.17 to -0.11)	< 0.001	-0.14 (-0.17 to -0.1)	< 0.001
Block 3 vs. Block 1	-0.2 (-0.24 to -0.17)	< 0.001	-0.2 (-0.23 to -0.17)	< 0.001
Movement number per block				
Block 2 vs. Block 1	0.003 (0.001 to 0.004)	< 0.001	0.003 (0.001 to 0.004)	< 0.001
Block 3 vs. Block 1	0.003 (0.002 to 0.004)	< 0.001	0.003 (0.002 to 0.004)	< 0.001
Number of valid movements per block (per valid movement)	-0.01 (-0.01 to – 0.01)	< 0.001	-0.01 (-0.01 to -0.01)	< 0.001
Age (per year)	0.006 (0.002 to 0.011)	0.003	0.006 (0.003 to 0.01)	0.001
Sex (female vs. male)	-0.03 (-0.09 to 0.02)	0.248	-0.03 (-0.08 to 0.02)	0.203
Oxygen saturation (per 1% increase)	-0.00 (-0.06 to 0.06)	0.991	-0.001 (-0.02 to 0.02)	0.928
Oxygen saturation, acetazolamide vs. placebo	0.03 (-0.04 to 0.1)	0.409	0.03 (0.01 to 0.05)	0.01
Oxygen saturation, 3100m vs. 760m	0.01 (-0.05 to 0.06)	0.79	0.01 (-0.01 to 0.03)	0.386
Oxygen saturation, effect on treatment effect	-0.06 (-0.13 to 0.02)	0.166	-0.06 (-0.08 to -0.03)	< 0.001
Constant	2.05 (-3.96 to 8.06)	0.503	2.11 (0.22 to 4.00)	0.029
Random effects				
ID	0.01 (0.01 to 0.01)		0.01 (0.01 to 0.01)	
Residual	0.11 (0.10 to 0.12)		0.11 (0.11 to 0.11)	

Sqrt = square root, DE = directional error at peak velocity

Supplementary Table S4: Logistic regression for movement validity

	**Post-sleep recall**		**Immediate recall**		**Adaptation 60°**	
	**Average marginal effect (95%CI)**	**p**	**Average marginal effect (95%CI)**	**p**	**Average marginal effect (95%CI)**	**p**
Acetazolamide vs. placebo	0.05 (-0.04 to 0.15)	0.257	0.02 (-0.06 to 0.1)	0.58	0.003 (-0.07 to 0.08)	0.938
3100 m vs. 760 m	0.05 (0.03 to 0.08)	< 0.001	-0.04 (-0.08 to 0.00)	0.08	0.002 (-0.01 to 0.02)	0.819
Treatment effect (Interaction drug-altitude)	0.36 (0.3 to 0.42)	< 0.001	0.01 (-0.07 to 0.08)	0.887	0.04 (0.01 to 0.07)	0.003
Movement number (per additional movement)	0.002 (0.001 to 0.003)	< 0.001	0.003 (0.002 to 0.003)	< 0.001	0.001 (0.000 to 0.002)	<0.001
Block						
Block 2 vs. 1	0.17 (0.15 to 0.19)	< 0.001	0.2 (0.18 to 0.22)	< 0.001	0.04 (0.02 to 0.05)	<0.001
Block 3 vs. Block 1	0.24 (0.22 to 0.26)	< 0.001	0.26 (0.24 to 0.28)	< 0.001	0.06 (0.04 to 0.07)	<0.001
Movement number, per block						
Block 2 vs. Block 1	-0.003 (-0.004 to – 0.001)	< 0.001	-0.003 (-0.005 to -0.002)	< 0.001	-0.001(-0.002 to 0.001)	0.272
Block 3 vs. Block 1	-0.004 (-0.005 to – 0.002)	< 0.001	-0.005 (-0.006 to -0.003)	< 0.001	-0.00 (-0.002 to 0.001)	0.572
Age (per year)	-0.01 (-0.02 to – 0.003)	0.003	-0.01 (-0.02 to -0.01)	< 0.001	-0.011 (-0.017 to -0.005)	<0.001
Sex (female vs. male)	-0.09 (-0.19 to -0.001)	0.048	-0.18 (-0.25 to -0.1)	< 0.001	-0.1 (-0.18 to -0.03)	0.009
Target direction (from left to right)						
Target 2 vs. 1			0.05 (0.03 to 0.07)	< 0.001	0.06 (0.04 to 0.08)	<0.001
Target 3 vs. 1			0.04 (0.02 to 0.06)	< 0.001	0.08 (0.05 to 0.1)	<0.001
Target 4 vs. 1			0.01 (-0.01 to 0.03)	0.279	0.06 (0.04 to 0.08)	<0.001
Lake Louise Score	-0.03 (- 0.04 to – 0.02)	< 0.001				
Sleep quality (VAS scale)	- 0.001 (-0.002 to -0.001)	< 0.001				
Mean arterial pressure (per 1 mmHg increase)			-0.004 (-0.005 to -0.003)	< 0.001		
Oxygen saturation (per 1% increase)	0.01 (0.01 to 0.02)	< 0.001	-0.006 (-0.013 to 0.002)	0.143		
Oxygen saturation in%, acetazolamide vs.placebo	0.08 (0.07 to 0.09)	< 0.001	-0.004 (-0.02 to 0.01)	0.574		
Oxygen saturation in%, 3100m vs. 760m	-0.01 (-0.02 to – 0.01)	0.001	0.013 (0.001 to 0.024)	0.028		
Oxygen saturation in %, effect on treatment effect	-0.09 (- 0.11 to – 0.08)	< 0.001	0.03 (0.01 to 0.05)	0.017		

VAS = visual analog scale
